# Context-dependent activity of A domains in the tyrocidine synthetase

**DOI:** 10.1038/s41598-019-41492-8

**Published:** 2019-03-26

**Authors:** Anna Degen, Florian Mayerthaler, Henning D. Mootz, Barbara Di Ventura

**Affiliations:** 1 German Cancer Research Center DKFZ and Faculty of Biosciences, University of Heidelberg, 69120 Heidelberg, Germany; 2 Department of Chemistry and Pharmacy, Institute of Biochemistry, University of Münster, 48149 Münster, Germany; 3 Institute of Biology II, University of Freiburg, 79104 Freiburg, Germany; 4 Signalling Research Centers BIOSS and CIBSS, University of Freiburg, 79104 Freiburg, Germany

## Abstract

Non-ribosomal peptide synthetases (NRPSs) are large, modular enzymes that produce bioactive peptides of tremendous structural and chemical diversity, due to the incorporation, alongside the canonical 20 amino acids, of non-proteinogenic amino acids, fatty acids, sugars and heterocyclic rings. For linear NRPSs, the size and composition of the peptide product is dictated by the number, order and specificity of the individual modules, each made of several domains. Given the size and complexity of NRPSs, most *in vitro* studies have focused on individual domains, di-domains or single modules extracted from the full-length proteins. However, intermodular interactions could play a critical role and regulate the activity of the domains and modules in unpredictable ways. Here we investigate *in vitro* substrate activation by three A domains of the tyrocidine synthetase TycC enzyme, systematically comparing their activity when alone (with the respective PCP domain), in pairs (di-modular constructs) or all together (tri-modular construct). Furthermore, we study the impact of mutations in the A or PCP domains in these various constructs. Our results suggest that substrate adenylation and effects of mutations largely depend on the context in which the domains/modules are. Therefore, generalizing properties observed for domains or modules in isolation should be done with caution.

## Introduction

**Figure 1:**
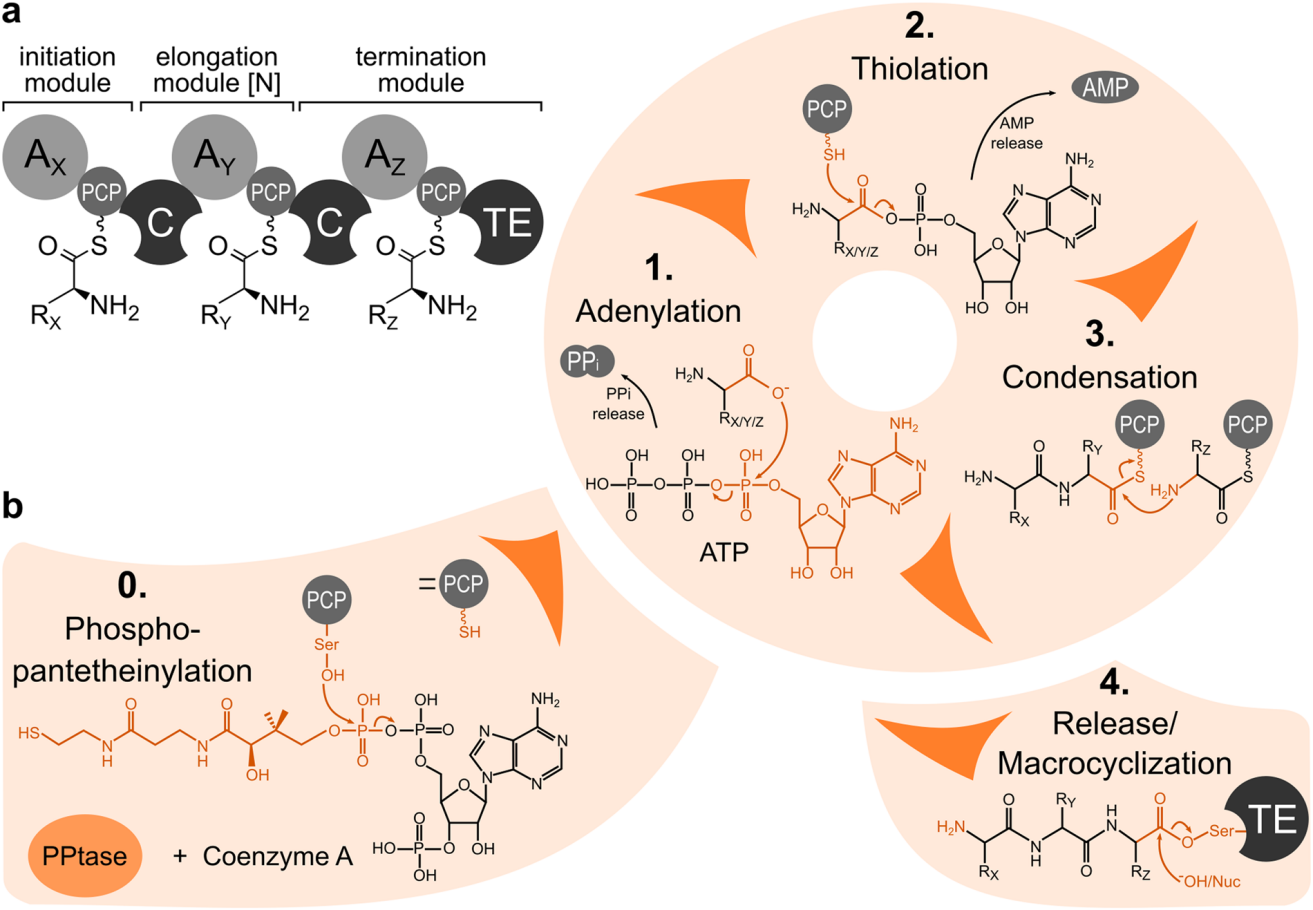
The biosynthetic cycle of NRP formation. (**a**) A standard NRPS consists of an initiation, one or more [N] elongation and a final termination module. Each module in turn consists of various domains, each playing a crucial role in the NRP formation process. A_X/Y/Z_: Adenylation domains specific for amino acids with side chains X, Y and Z, respectively; PCP: Peptide Carrier Protein domain; C: Condensation domain; TE: Thioesterase domain. (**b**) Overview of chemical reactions prior to (0. Phosphopantetheinylation), during (1. Adenylation, 2. Thiolation and 3. Condensation) and at the end of (4. Release/Macrocyclation) the biosynthetic cycle of NRP formation performed by an external PPtase, A, PCP, C and TE domains, respectively. Chemical moieties involved in or passed on during the reaction are shown in orange. For a better overview, NRPS domains involved in the reactions are omitted in this scheme, except for the small PCP domain.

Non-ribosomal peptide synthetases (NRPSs) are modular mega-enzymes that produce peptides independently of the ribosome. For linear NRPSs, the size and composition of the non-ribosomal peptides (NRPs) they produce is entirely dictated by the number, specificity and order of modules they are made of^1^. A basic NRPS module is typically composed of an adenylation (A) domain, a peptidyl carrier protein (PCP) domain, and a condensation (C) domain (Fig. 1a). The A domain consists of a large N-terminal and a small C-terminal subdomain, which act together to specifically recognize the substrate and activate it through the attachment of adenosine monophosphate (AMP) to the C-terminal hydroxyl group (Fig. 1b, 1.). Upon successful substrate adenylation, the small subdomain rotates about 140° to proceed to the thiolation state, accompanied by the release of pyrophosphate (PP_i_)^2^. Some A domains require additional proteins called MbtH^3^ or MbtH-like proteins (MLP)^4^ for proper function and stability. The activated building block is then attacked by the thiol group of the phosphopantetheinyl (PPE) arm (Fig. 1b, 2.) post-translationally added to a conserved serine within the PCP domain by an external PPtase^5^ (Fig. 1b, 0.). After thiolation, the PCP domain passes the substrate on to the C domain, where the upstream and downstream building blocks are fused via peptide bond formation^6,7^ (Fig. 1b, 3.). In this way, the non-ribosomal peptide grows from module to module in an assembly line fashion until the final product is released by a release module, e.g. a thioesterase (TE) domain^8^ (Fig. 1b, 4.).

The attractiveness of NRPs for biomedical and biotechnological applications lies in their exceptional chemical and structural diversity, which is associated with the likelihood of finding peptides active in a biological process of interest (e.g. bacterial or viral replication). This diversity is achieved in numerous ways, some of which include: (i) A domain specificity is not limited to proteinogenic amino acids; (ii) initial C domains can incorporate fatty acids; (iii) auxiliary domains can modify (e.g. epimerize, oxidize, methylate, just to name a few) the building blocks along the way and (iv) the TE domain can release the product as a cyclic peptide. Understanding how NRPSs work is important if we wish to exploit them for synthesizing custom NRPs with novel bioactivities^9^, like demonstrated in few successful approaches^10–12^.

During the synthesis process, NRPSs have to undergo large conformational changes to consent to the growing peptide chain to travel along the modules. From studies carried out on individual modules extracted from multi-modular NRPSs, the A domain was reported to adopt three different conformations: (i) an open conformation, where no ligand is bound; (ii) an adenylation conformation, where the small C-terminal A_sub_ domain is reconfigured by ~48° to complex with ATP and the substrate to perform the adenylation reaction; and, finally, (iii) a thiolation state, in which the C-terminal A_sub_ domain is rotated by ~140° around the large N-terminal A_core_ to tether the activated substrate to the PPE arm of the PCP domain^13–16^. This A domain alternation mechanism is expected to contribute most to the NRP elongation process. At the same time, the PCP domain likely also undergoes some movements as it needs to span the distance between the upstream C domain, the A domain and the downstream C or TE domain, which cannot be solely attributed to the length of the PPE arm (~20 Å) or to the A_sub_ domain movement^17^. The C domain has also been proposed to exist in an open and a closed state depending on substrate binding^18^, but the influence of these two states on the overall catalytic cycle of NRPSs remains elusive.

Insights into the conformational states of NRPSs have been obtained from crystal structures of individual domains as well as multi-domain constructs trapped in a specific state by point mutations^17^ or mechanism-based inhibitors^19–22^. Thus, each structure represents a staged snapshot of the synthesis process, but the overall movie has yet to be shot. NMR studies are capable of analyzing protein conformational changes in solution, but at least the traditional methods are limited to relatively small proteins and therefore only individual PCP and TE domains or PCP-TE di-domains have been analyzed this way^23–25^. Recently, negative stain electron microscopy (EM) images of an A-PCP-C tri-domain construct in complex with a MbtH-like protein (MLP) were compared to a crystal structure of the same protein complex^22^. The EM images supported the notion that domains within a NRPS module adopt many conformations and do not seem to exist in a set of well-defined states^26^, in contrast to what was previously proposed^27^.

Due to technical limitations and the challenge to express and purify such large proteins, mechanistic studies on multi-modular constructs are lacking. Thus, it is to date unclear how individual modules are structurally and functionally embedded within the context of full-length NRPSs or truncated variants thereof consisting of more than one module. It is licit to ask whether there is an intrinsic communication between modules that ensures proper directionality and progression of NRP synthesis and peptide identity.

We specifically asked ourselves whether the adenylation activity of A domains would depend on the context they are embedded in and whether mutations directed towards them or their associated PCP domains would have the same effect regardless of the presence or state of neighboring modules.

**Figure 2:**
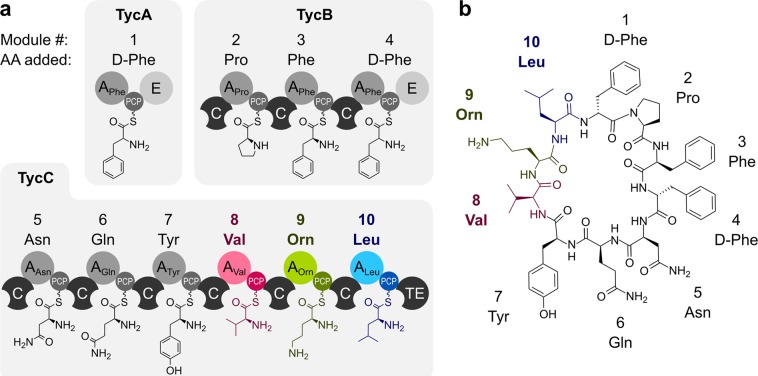
The tyrocidine synthetase produces the antimicrobial cyclic peptide tyrocidine. (**a**) Schematic depiction of the three enzymes that make up the tyrocidine synthetase. Numbers refer to the modules. Modules used in this study are shown in color. Amino acids are depicted attached to the PPE arms. (**b**) Chemical structure of tyrocidine. The three amino acids incorporated by the modules used in this study are shown in color. Colors as in (**a**).

To this aim, we constructed a library of 34 constructs based on the very well-studied tyrocidine synthetase TycC (Fig. 2), which can be expressed in *E*. *coli* without any further codon optimization and without co-expression of a MbtH-like protein. Alongside individual A-PCP di-domain constructs consisting of the last three A-PCP di-domains of TycC (TycC8, TycC9 and TycC10, respectively), we built two di-modular constructs (TycC8-9 and TycC9-10) and one tri-modular construct (TycC8-9-10). All these constructs were either kept “wild type” or mutated in each A and PCP domain (one at a time). To systematically compare A domain activity we employed a PP_i_ release assay^28^ which has been recently adapted to the study of NRPSs^29–31^. The advantage of this assay is that the detection is spectroscopic and does not require radioactivity. Our findings suggest that the context into which the A domains are embedded does play a critical role in regulating their activity, likely because modules in NRPSs interact in complex ways that are not easy to predict by knowing the structure and function of the individual components.

## Results

### Analysis of A-PCP di-domains

**Figure 3:**
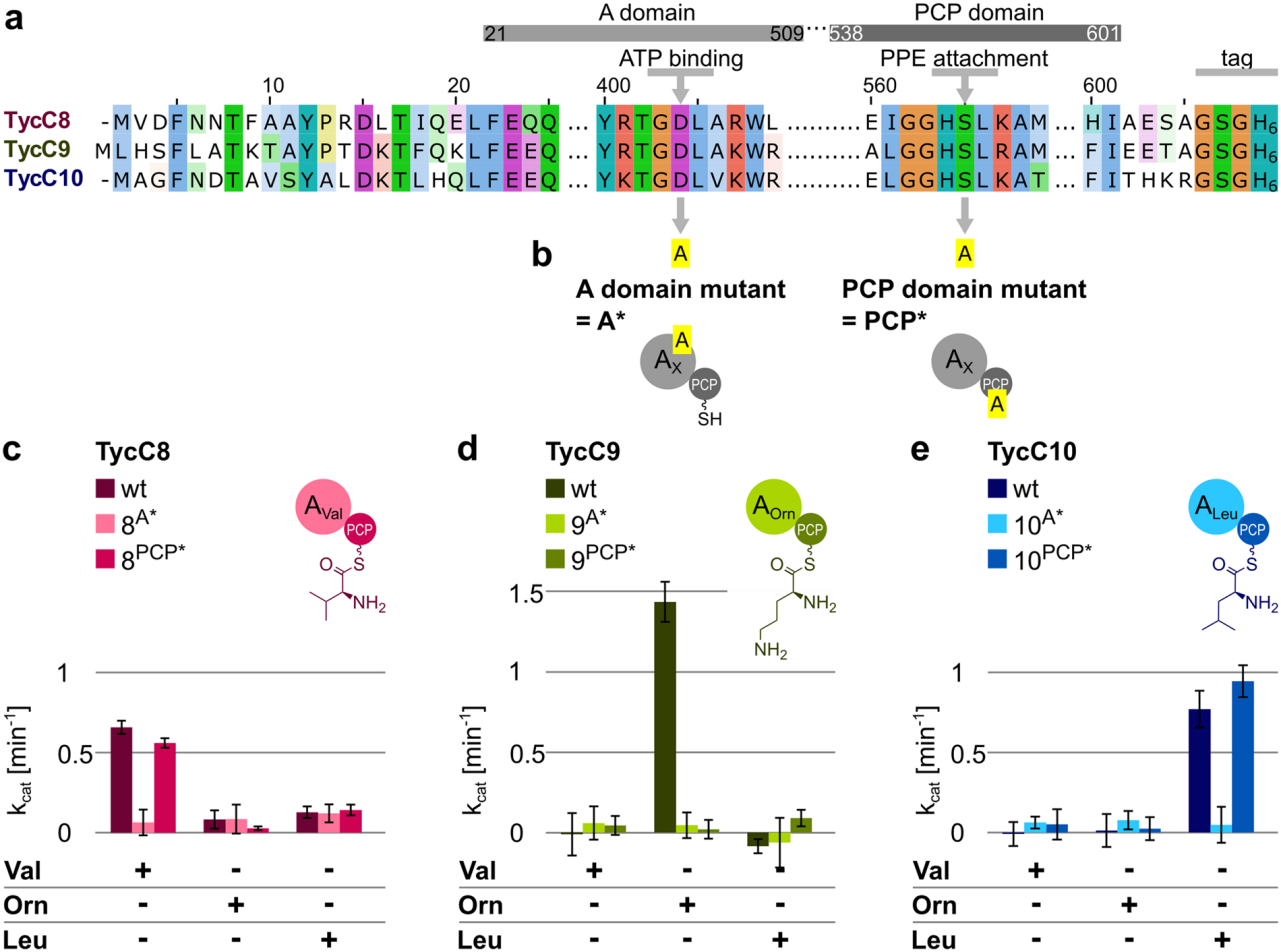
Activity of selected A domains within “wild type” or mutated A-PCP di-domain constructs of TycC. (**a**) Sequence alignment of the indicated constructs. Amino acids are color-coded according to their category and degree of conservation, following Clustal X coloring scheme. Numbers above the sequence indicate the amino acid position where 1 is set as the beginning of construct TycC8. Predicted boundaries for A and PCP domains are shown above the sequences. The residue critical for ATP binding and the serine needed for PPE arm attachment are pointed at by grey arrows. “Tag” indicates a GSG linker plus the 6x His tag. (**b**) Position and identity of mutations used to inactivate the A and PCP domains. (**c–e**) Upper panel: schematic depiction of construct with amino acid attached to the PPE arm. Colors as in Fig. 2a. Lower panel: bar graph showing k_cat_ values for the constructs in the upper panel in the presence of the indicated amino acids, calculated using the online PP_i_ release assay after subtraction of the background (=no substrate) value. Amino acids were added at 1 mM, enzymes at 0.5 µM. Data represent the mean (±standard deviation, SD) of three independent experiments.

We first used the Protein Families Database Pfam^32^ to locate the A and PCP domains of the last three modules in TycC (Fig. 3a). For each A domain we tested two slightly different start sites, of which one was previously established^33^ (Fig. S1a). The corresponding six A-PCP di-domain constructs were amplified from the genome of *Brevibacillus brevis ATCC 8185* and cloned with a C-terminal His-tag, expressed in BAP1 cells^34^ and purified in batch using Ni^2+^ agarose beads. We refer to these constructs as TycC8 ((1) and (2)), TycC9 ((1) and (2)) and TycC10 ((1) and (2)). As BAP1 cells express the chromosomally integrated promiscuous PPtase Sfp^35^, we expect the PCP domains in the purified proteins to be loaded with the PPE arm, as previously reported^36,37^. Interestingly, we found that with the new start site identified in this study the adenylation activities of the A domains were more comparable, while the previously established start site led to higher variability (Fig. S1b). For this reason, we decided to carry on further studies with the new start site (1).

To analyze the effect of inactivating the A domain, we introduced a point mutation changing the aspartic acid of the core motif A7 (Y[R/K]TGDL)^38^ to alanine (D→A, Fig. 3b). This mutation dramatically decreases the affinity of the A domain for ATP^39,40^. Since ATP is needed to adenylate the substrate, this mutation effectively results in lack of amino acid activation by the A domain. We refer to these constructs as TycC8^A*^, TycC9^A*^ and TycC10^A*^ (Fig. S2a,d,g). To analyze the effect of inactivating the PCP domain, we introduced a point mutation changing the serine within the conserved consensus sequence [I/L]GG[D/H]SL^41^ to alanine (S→A, Fig. 3b). The serine is needed as attachment point for the PPE arm^42^; thus, this mutation effectively results in a PCP domain that cannot carry the amino acid. We refer to these constructs as TycC8^PCP*^, TycC9^PCP*^ and TycC10^PCP*^ (Fig. S2a,d,g). The constructs were expressed in and purified from *E*. *coli* cells (Fig. S2b,e,h); we then investigated wild type and mutated constructs using the online PP_i_ release assay^30^. Note that for the detection of the absorbance at 340 nm we use a plate reader compatible with high-throughput measurements. This means we cannot measure few discrete activation cycles by the A domain, but rather repeated activation.

The native A domains showed the expected specificity (Fig. 3c–e), even when the natural substrates were mixed with other amino acids (Fig. S2c,f,i). Adding the natural TE domain to TycC10 did not change the results (Fig. S2j,k). Mutating the A domain resulted in an almost complete abrogation of adenylation activity. PCP domain mutation did not affect the activity of the A domains in TycC8^PCP*^and TycC10^PCP*^ (Fig. 3c,e), while it strongly affected the activity of the A domain in TycC9^PCP*^ (Fig. 3d). There have been reports of A domains analyzed in the absence of the accompanying PCP domain showing that they are active^43,44^. TycC8^PCP*^and TycC10^PCP*^are in line with these observations. It is all the more intriguing that the same mutation so strongly affects the activity of the A domain in TycC9.

To gain some mechanistic insights into why the PCP domain so greatly influences the substrate adenylation rate of TycC9, we measured A domain activity by TycC9 and TycC9^PCP*^ in the presence of lysine. Indeed, it has been previously reported that the ornithine thioester – formed once ornithine is attached to the PPE arm – undergoes intramolecular cyclization with subsequent release from the NRPS^33^. This likely accelerates the rate at which the A domain can proceed to a new round of adenylation compared to the case in which the PCP domain is mutated, no PPE arm is attached and, consequently, no cyclization and release occur. Lysine, on the other hand, does not undergo such intramolecular cyclization. Therefore, if TycC9 and TycC9^PCP*^ had similar activities with lysine, we could infer that ornithine cyclization is the reason why the PCP domain mutation impacts so much the activity of the A domain for ornithine. Despite showing both very low signals in the online PP_i_ release assay, TycC9 and TycC9^PCP*^ had similar activities with lysine (Fig. S2l). This was even more evident when comparing TycC9 and TycC9^PCP*^ constructs with start site (2), since in this case lysine was accepted more robustly (Fig. S2l).

### Analysis of A domains embedded in di-modular constructs

**Figure 4:**
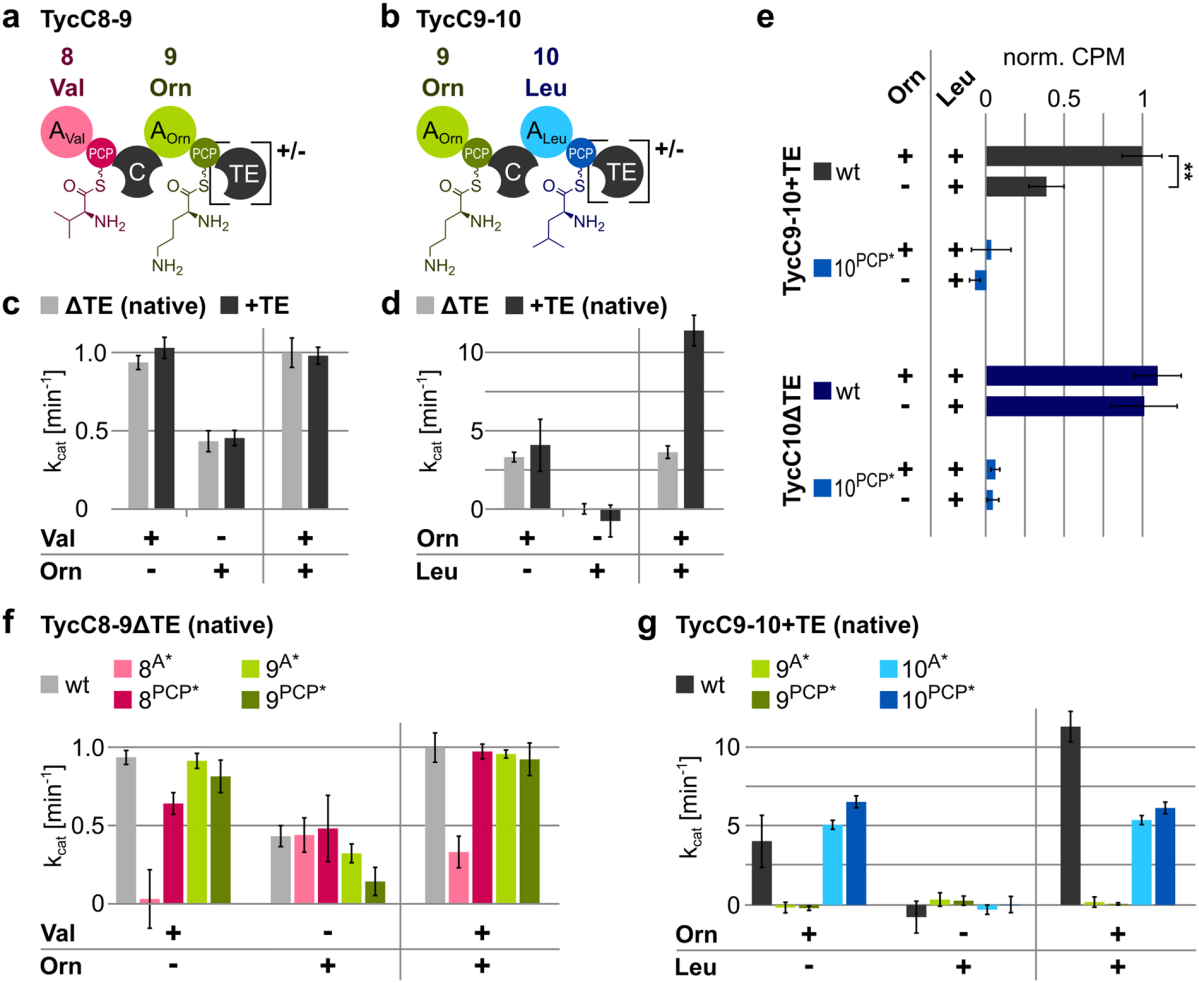
Activity of selected A domains within “wild type” or mutated di-modular constructs of TycC. (**a**,**b**) Schematic depiction of constructs with amino acids attached to the PPE arms. Brackets highlight the domain that is either present (+) or absent (−). Colors as in Fig. 2a. (**c**,**d**,**f,g**) Graphs showing k_cat_ values for the indicated constructs in the presence of the indicated amino acids, calculated using the online PP_i_ release assay after subtraction of the background (=no substrate) value. Amino acids were added at 1 mM, enzymes at 0.5 µM, with the exception of TycC9-10ΔTE, which was added at 0.25 µM. Data represent the mean (±standard deviation, SD) of three independent experiments. (**e**) Thiolation assay with [^3^H]-labelled L-Leu in the presence or absence of Orn. Counts per minute (CPM) were normalized to the value for TycC9-10 + TE wt. **p < 0.01. Data represent the mean (±standard deviation, SD) of three independent experiments.

Using the boundaries for the A and PCP domains identified using Pfam as described above (start site (1)), we then constructed two di-modular constructs: one consisting of modules 8 and 9 (we refer to this construct as TycC8-9) and one containing modules 9 and 10 (which we refer to as TycC9-10) (Fig. 4a,b). Note that, naturally, module 10 contains the TE domain for release of the final peptide product (Fig. 2a). Therefore, we decided to create a variant of TycC8-9 artificially containing the same TE domain of TycC9-10 (Fig. 4a), as well as a TycC9-10 variant where the TE domain was removed (Fig. 4b). Like this, we have both constructs with and without terminal TE domain. The four constructs were expressed in and purified from *E*. *coli* cells (Fig. S3a,b), then analyzed adding the amino acid substrates in various constellations using the online PP_i_ release assay.

TycC8-9, with and without TE domain, showed activation of valine and ornithine, regardless whether the amino acids were introduced separately or simultaneously (Fig. 4c). The presence of leucine, substrate for module 10, did not alter these results (Fig. S3c). Moreover, the same was observed when lysine was used instead of ornithine. Interestingly, for this construct, the activation rate for lysine was higher than that for ornithine (Fig. S5a). These data support the notion that A domains in elongation modules activate their substrates even when upstream A domains do not.

TycC9-10, with and without TE domain, on the other hand, behaved in an unexpected way. While ornithine given as only substrate was activated, leucine was not when present as the only amino acid in the reaction (Figs 4d and S4a). We observed this also when lysine was used instead of ornithine (Fig. S5b), as well as when leucine was mixed with valine (Fig. S3d). When both amino acids were present, however, the adenylation rate was higher than that measured for ornithine alone, at least for TycC9-10 + TE (Fig. 4d). The same occurred when adding also valine to the mixture (Fig. S3d). Taken together, these data suggest that the A domain for leucine, when embedded in this specific di-modular construct, is unable to repeatedly activate its own substrate unless the upstream module does so as well. To confirm this interesting finding and to exclude the possibility that failed leucine activation were due to an inherent dysfunction of this construct, we performed a thiolation assay with TycC9-10 + TE (Fig. 4e). In this assay, we measured the amount of radioactively labelled leucine which gets covalently attached to the PPE arm of the enzyme of interest. TycC10 served as a positive control, while its PCP mutant (TycC10^PCP*^) and the PCP mutant of module 10 in the di-modular TycC9-10 + TE construct (TycC9-10^PCP*^) served as negative controls. In agreement with the k_cat_ values for TycC9-10 + TE obtained in the online PP_i_ release assay, the radioactive signal when ornithine and leucine were present was as high as that for the positive control (Fig. 4e), demonstrating that thiolation effectively occurs in this case. In contrast to the result of the online PP_i_ release assay, the signal obtained when solely leucine was added was well above background. This suggests that TycC9-10 + TE does activate leucine, but not repeatedly (compare Fig. 4,d,e).

As previously mentioned, TycC9-10 was also largely impacted by removal of the TE domain, as seen when both amino acids were present at the same time (Fig. 4d). A reason for this could be that the TE domain helps releasing the di-peptide, which is exclusively formed when both amino acids are present, thus allowing the A domains to get on with the next activation cycle faster. Using mass spectrometry, we confirmed production of the di-peptide by TycC9-10 (Fig. S6a).

We then studied the effect of mutating each A and PCP domain individually in the TycC8-9ΔTE “native” construct (i.e., without additional TE domain). The mutants were expressed in and purified from *E*. *coli* cells with good yields (Fig. S3e). Mutation of the A domain in module 8 resulted in no activity when valine was fed to the enzyme. Mutation of the PCP domain in module 8 had also an effect (Fig. 4f). These results are generally in line with what was observed when analyzing the same mutations on TycC8 (Fig. 3c), albeit the PCP mutant in the context of the di-modular construct is more impaired than the same mutant in TycC8 (31% decrease of the activity (p < 0.005) versus 15% decrease (p = 0.016), respectively). Mutation of the A domain in module 9 had only a minor effect on the activation of ornithine (Fig. 4f). This is in striking contrast with the results obtained with this very mutation on TycC9 (Fig. 3d). Mutation of the PCP domain in module 9 also decreased the activity of the A domain (Fig. 4f), as previously seen for this mutation on TycC9 (Fig. 3d). To test whether ornithine cyclization is the reason for the effect of the PCP mutation also in this context, we performed the online PP_i_ release assay feeding lysine instead of ornithine, alone and together with valine (Fig. S5a). We found that, in this case, the mutant behaves like the wild type, confirming what we found with TycC9 (see Fig. S2l). Surprisingly, we also observed that lysine was now accepted as substrate even better than ornithine itself (compare Figs S2l and S5a). These data suggest that the presence of upstream elements changes the specificity of the A domain in module 9.

We also studied the mutations on the A and PCP domains in the TycC9-10 + TE “native” construct (i.e., with natural TE domain). These mutants, as well as the wild type protein, were purified from *E*. *coli* cells with lower yields compared to the other constructs (Fig. S3f). Mutating the A and PCP domains in module 9 resulted in no activity when ornithine was presented to the enzyme (Fig. 4g), in good agreement with the results obtained for these mutations on the di-domain construct TycC9 (Fig. 3d). The PCP mutation (9^PCP*^) had, moreover, a similar negative impact also on TycC9-10ΔTE (Fig. S3d). Unfortunately, mutations on module 10 are hard to assess, as the enzyme does not repeatedly activate leucine unless ornithine is present. Therefore, the effects of the mutations are only evident in the reaction in which both substrates are given (Fig. 4g). However, with the online PP_i_ assay, it is not possible to distinguish between the activity of the individual A domains in the enzyme, making it impossible to study the effects of the mutations to module 10 independently from the activity of module 9 in TycC9-10. Given the results of the thiolation assay, though, we can at least conclude that the PCP mutation has the expected effect of preventing thiolation of leucine (Fig. 4e).

### Analysis of A domains embedded in a tri-modular construct

**Figure 5:**
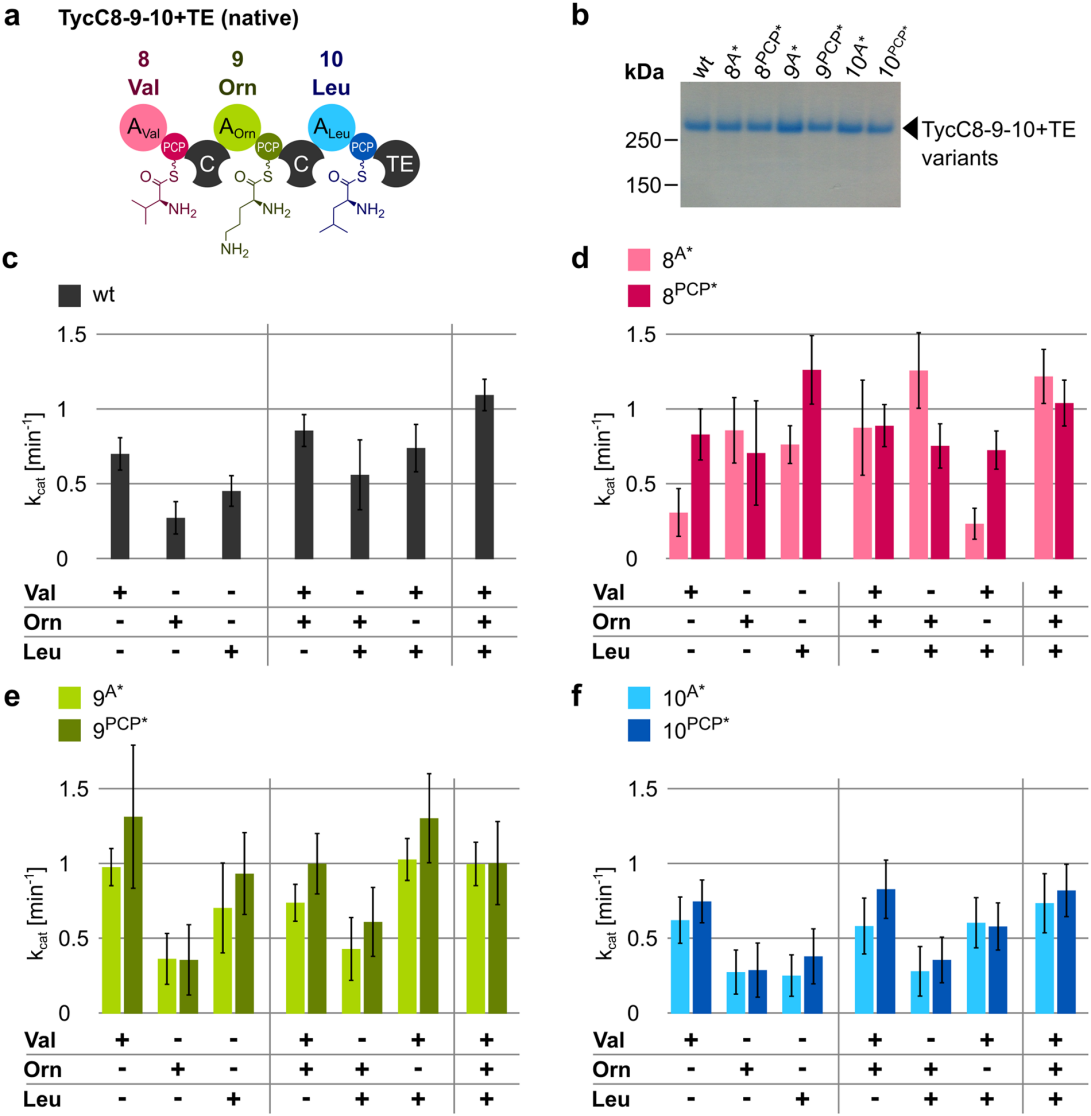
Activity of selected A domains within the “wild type” or mutated tri-modular construct of TycC. (**a**) Schematic depiction of the construct with amino acids attached to the PPE arms. Colors as in Fig. 2a. (**b**) Coomassie-stained SDS-gel showing the indicated purified proteins. (**c**–**f**) Graphs showing k_cat_ values for the indicated constructs in the presence of the indicated amino acids, calculated using the online PP_i_ release assay after subtraction of the background (=no substrate) value. Amino acids were added at 1 mM, enzymes at 0.5 µM. Data represent the mean (±standard deviation, SD) of three independent experiments.

Finally, we built the tri-modular construct encompassing modules 8, 9 and 10 plus the native TE domain (Fig. 5a). We refer to this construct as TycC8-9-10 + TE. The enzyme could be expressed in and purified from *E*. *coli* cells with good yields despite its very large size of about 326 kDa (Fig. 5b). It was very intriguing to see that, in this context, the A domain of module 10 activated leucine when present as only substrate (Figs 5c and S4b), contrary to the behavior of this module in the di-modular construct TycC9-10 (Fig. 4d,g). As expected, the other two amino acids were also activated. Activation occurred regardless whether the amino acids were given singularly, in pairs or all together. TycC8-9-10 + TE also accepted lysine in place of ornithine (Fig. S5c). The fact that the k_cat_ values are higher when all three substrates are given suggests that the tri-peptide may be made. This was confirmed by mass spectrometry (Fig. S6b).

We introduced the same A and PCP mutations as before to each module of TycC8-9-10 + TE individually and purified these mutants (Fig. 5b). Surprisingly, for all constructs, the mutation to the A domain did not abolish substrate adenylation (Fig. 5d–f), as seen for the same A domains in other settings (Figs 3c–e and 4f,g). Moreover, mutating the PCP domain of module 9 in this context had practically no effect on the adenylation activity of the A domain (Fig. 5e), contrary to the same mutation in the context of the di-domain TycC9 construct (TycC9^PCP*^; Fig. 3d) and the di-modular TycC8-9 and TycC9-10 constructs (TycC8-9^PCP*^ and TycC9^PCP*^-10, respectively; Fig. 4f,g). However, we could not detect the tripeptide for the tri-modular construct mutated in the A domain of module 9 (Fig. S6c,d). The tri-modular construct mutated in the PCP domain of module 9 was used as negative control, while the construct without mutations as positive control.

## Discussion

NRPSs are fascinating enzymes that attract the attention of scientists from different disciplines due to the enormous benefit that would derive from the ability to re-engineer them to produce desired peptides. While exciting advances have been recently made towards finding rules to cut and paste modules in a defined manner to build functional chimeric NRPSs^11,12^, it remains to be seen if such rules are truly generally applicable. Therefore, detailed biochemical characterization of NRPSs is still needed to understand their mechanism of action to a greater degree. It is, for instance, still rather unclear how modules in a multi-modular NRPS communicate with one another, as most studies are carried out with single domains or modules, extracted from the larger proteins they belong to. However, peptides produced by engineered NRPSs, with potentially interesting new functionalities, should likely consists of several amino acids. Thus, it is important to move the focus towards the study of NRPS domains and modules in larger contexts rather than as isolated entities. In this work, we have done exactly this: we have explored how “the context” influences the behavior of individual domains and entire modules. While we cannot provide a mechanistic explanation for all the acquired data, we reckon they do support the conclusion that the behavior of a NRPS domain/module is highly dependent on the presence and status of upstream and downstream elements (what we call “the context”).

There are three sets of data pointing to this conclusion derived from measuring A domain activity for different constructs with different substrate combinations in otherwise identical conditions: (i) effects of mutations; (ii) specificity of A domains; and (iii) activity of A domains.

Mutating the ATP binding site of the A domain has the expected effect of abolishing adenylation only when applied to the excised A-PCP di-domains (Fig. 3c–e). However, when applied to multi-modular constructs, the same mutation has an effect only when applied to the A domain of the starting module (Figs 4f,g and 5d–f). One possible explanation for this is that the binding of ATP, that should be strongly affected by the absence of the critical aspartic acid of the core A7 motif, gets stabilized by neighboring structural elements, which are not present for starter A domains that lack an upstream module/domain. However, given that we were not able to detect the tripeptide that should have been produced by one of the mutant tri-modular constructs (TycC8-9^A*^-10 + TE), it is likely that the mutation nonetheless affects other aspects of A domain functionality, such as successful communication with the PCP domain. Indeed, while we cannot exclude that the tripeptide is made at levels below those we can detect under these conditions, the aspartic acid we mutated is conserved in all A domains^40,45^, regardless if starter or elongation ones, indicating that a compensation of the mutation supporting peptide production is unlikely. Another explanation is that the adenylation activity we measure is that of another A domain present in the multi-modular construct. As a matter of fact, the values obtained in the online PP_i_ assay cannot be assigned to any specific A domain. This would suggest that the point mutation changes the specificity of neighboring A domains, for instance due to a different conformation assumed in the absence of ATP but presence of the substrate^16^. The current experimental techniques at our disposal do not allow us to clarify this issue.

Mutating the PCP domain has also very context-dependent effects. In the di-domain constructs, the mutation does not interfere with the activity of the A domain, when considering lysine instead of ornithine as substrate for TycC9 (Figs 3c,e and S2l). This supports the notion that the A domain can activate the substrate – accompanied by the release of PP_i_, which we measure – independent of the next step, which is its attachment onto the PPE arm. Indeed, other studies have used excised A domains where the PCP domain was absent all together and they could measure A domain activity, albeit it proved to be lower than in the presence of the PCP domain^46^. However, the same PCP domain mutation impairs the activity of the cognate A domain in the di-modular constructs (Fig. 4f,g). Notably, the degree of impairment itself depends on the context, as the activity of the A domain of module 8 is less affected than that of the A domain of module 9 (32% decrease versus 67% decrease, respectively). Moreover, in our study, we have the advantage to be able to compare the very same module (module 9), when serving as starting (TycC9-10) or terminating (TycC8-9) module. Interestingly, the same mutation to the PCP domain of module 9 has a much more dramatic effect when this module is the starting one (compare Fig. 4f,g). In this case, using lysine instead of ornithine does not change the result (Fig. S5b), suggesting that here the effect of the mutation is unrelated to ornithine cyclization as seen when module 9 is the terminating one (Fig. S5a). Unexpectedly, the mutations have no effect on substrate adenylation by the cognate A domain in the tri-modular construct (Fig. 5d–f).

Not only the mutations, but also the acceptance of a certain substrate is influenced by the context: changing the start site affected the degree by which the di-domain TycC9 construct accepted lysine (Fig. S2l); this was seen also when module 9 was part of larger constructs (Fig. S5). Our data on substrate specificity modulation by upstream elements are in good agreement with what was recently proposed by Meyer and colleagues^47^.

Finally, whether a module will repeatedly activate its own substrate also seems to depend, in certain cases at least, on the state of the upstream module(s). This is exemplified by the inability of TycC9-10 to repeatedly activate leucine in the absence of ornithine (Fig. 4d). This is not seen for TycC8-9, which can activate ornithine in the absence of valine (Fig. 4c), as well as in the tri-modular construct, where every substrate is activated when alone in the reaction (Fig. 5c). We speculate that there are some NRPSs that adopt conformations whereby the A domain is locked after two activation cycles (one to load the substrate onto the PPE arm and one additional cycle in which the substrate remains on the A domain) unless the condensation domain is actively condensing donor and acceptor substrates. Similar observations were made recently in another study^30^, however we would not generalize such a statement as we see that this does not happen all the time.

In conclusion, we believe that generalizations for domain and modules of NRPSs should be done with great caution, as their behavior appears to be very much dependent on the context they are embedded into.

## Methods

### Plasmid design, construction and site-directed mutagenesis

All plasmids used in this study were constructed via isothermal DNA assembly^48^ using the Gibson Assembly® Master Mix (NEB, Ipswich, MA, USA) according to the manufacturer’s instructions. The pTrc99a vector^49^ (hybrid trp/lac promoter; IPTG inducible; pBR322 origin of replication, amp^r^) served as a backbone for the insertion and expression of the different constructs. In short, the backbone and TycC inserts were amplified using the Phusion Flash High-Fidelity PCR Master Mix (Thermo Fisher Scientific, Waltham, MA, USA) from the full-length vector and a glycerol stock of *Brevibacillus Brevis* ATCC 8185 (kindly provided by Mohamed A. Mahariel, University of Marburg, Germany), respectively. In TycC9, specific for Orn, we detected several SNPs in comparison to the genomic sequence deposited in the NCBI database^43^. A domain: G190(ggg) → E(gag) and T201(acc) → I(atc). PCP domain: L35(ctt) → I(att). C domain: V6(gtc) → V(gtt) silent, V43(gta) → V(gtg) silent and A229(gcg) → E(gag). All primers (Sigma Aldrich) were designed for the DNA fragments to have an overlap between 15 and 25 base pairs which serve as a complementary recognition and fusion site during Gibson Assembly (Table S1). Once assembled, plasmids were transformed into chemically competent TOP10 cells (Thermo Fisher Scientific) and selected on LB agar plates containing 0.1 mg/ml ampicillin (Sigma Aldrich, Merck KGaA, Darmstadt, Germany) at 37 °C overnight. Plasmids were extracted from the cells and the cloning was verified by DNA sequencing (GATC, Eurofins Scientific, Konstanz, Germany). Once the desired plasmids were obtained, point mutations in the A and PCP domains were introduced as described before^50^. Sequencing confirmed the exclusive introduction of the desired mutations.

### Boundary selection

For single and di-modules ending after the PCP domain, a termination site was chosen 4 aa downstream of the respective PCP domain^51^. For constructs including the TE domain, the native termination site was chosen. The fusion site for introduction of the TE domain to the di-module TycC8-9 + TE construct was placed 4 aa downstream of the PCP9 domain, while the native PCP10-TE linker was shortened by 4 aa at the N-terminus. A GSG linker followed by a 6xHis tag was added to the C-terminus of all constructs.

### Protein expression and purification

Plasmids harboring the desired TycC constructs were transformed into BAP1 cells for protein expression and *in vivo* post-translational PPE attachment. BAP1 cells are derived from BL21(DE3) with the promiscuous PPtase Sfp integrated into their genome^34^ (a kind gift of Blaine Pfeifer, Department of Chemical and Biological Engineering, University of Buffalo, USA). A large expression culture (250–400 mL of LB-Miller containing 0.1 mg/mL ampicillin) was inoculated by adding 1:500 dilution of overnight culture and grown at 37 °C at 180 rpm until it reached an OD_600_ of 0.7–0.9. IPTG (Isopropyl-β-D-thiogalactopyranosid, Sigma Aldrich) was then added to the culture to a final concentration of 0.5 mM, and the culture was further grown at 18 °C overnight. The next morning, cells were harvested via centrifugation at 5 000 × g for 30 minutes. The cell pellet was resuspended in lysis buffer (50 mM HEPES, 12.5% glycerol, 500 mM NaCl, 5 mM imidazole, pH 7.5) and stored at −80 °C for 12 or more hours. Cells were lysed via sonication (100% power, 3 min, 30 s intervals, 6/10 s pulses) and cleared at 15 000 × g for 30 min. The supernatant containing the soluble protein fraction was incubated with 1 mL Ni-NTA beads (BioRad, Hercules, CA, USA) for 2 hours at 4 °C. The loaded Ni-NTA beads were washed twice with three column volumes of lysis buffer via gravity flow before being eluted with elution buffer (50 mM HEPES, 12.5% glycerol, 500 mM NaCl, 250 mM imidazole, pH 7.5). Eluate fractions were checked for protein content via Ponceau S staining. The fraction containing the major part of the protein was concentrated and buffer exchanged to storage buffer (50 mM Sodiumphosphate buffer, 20% glycerol, pH 7.25) in spin columns (Amicon® Ultra, Merck, Darmstadt, Germany) with the appropriate MWCO. Proteins were stored at −20 °C until use.

### A domain activity

The online PP_i_ release assay was performed as described before by Kittilä *et al*.^30^. In short, all components (Table 1) were combined in 50 mM TRIS-HCl, pH 7.5, and distributed in a 96 well plate, into which amino acids were previously pipetted. The absorption at 340 nm was continuously recorded for about 30 mins. k_cat_ values were calculated in Excel using the following formula:

$${k}_{{\rm{cat}}}=(\frac{{{\rm{\Delta }}\mathrm{OD}}_{{\rm{340}}}\,{{\rm{\min }}}^{-{\rm{1}}}}{{{\rm{\varepsilon }}}_{340}({\rm{NADH}})\ast {\rm{d}}\ast {c}_{{\rm{enzyme}}}\ast 2})$$

where ΔOD_340_ (in min^−1^) is the decrease in absorption at 340 nm calculated fitting the curve in the linear range; ε_340_(NADH) is the molar extinction coefficient of NADH (ε_340_(NADH) = 6 220 M^−1^ cm^−1^)^52^; d is the path length (~0.2 cm in a well of a 96 well plate filled with 100 µL reaction volume); and c_enzyme_ is the enzyme concentration (in µM).

**Table 1:** List of enzymes and other compounds used in the online pyrophosphate release assay.

Components (Sigma Aldrich unless otherwise indicated)	C_stock_	C_final_
D-Fructose 6-phosphate disodium salt hydrate	300 mM	3 mM
Fructose-6-phosphate Kinase, Pyrophosphate-dependent from Propionibacterium freudenreichii (shermanii)	20 U/mL	0.1 U/mL
Aldolase from rabbit muscle	100 U/mL	1 U/mL
Triosephosphate Isomerase from rabbit muscle	2500 U/mL	5 U/mL
α-Glycerophosphate Dehydrogenase from rabbit muscle	500 U/mL	5 U/mL
ATP	0.1 M	0.5 mM
MgCl_2_	100 mM	2 mM
EDTA	50 mM	0.1 mM
TycC xxx	20–100 µM	0.5 mM
ß-NADH (Gerbu Biotechnik)	100 mM	0.8 mM
Amino acid(s): V, O, K, L	0.1 M	1 mM

For each enzyme-substrate combination, the mean k_cat_ value of three measurements was calculated and background subtracted by the mean value of the negative control. The error was determined by subtracting the standard deviation (SD) of the negative control from the SD of sample, including error propagation:

$${\rm{SD}}\,({k}_{{\rm{cat}}})=\sqrt{{{\rm{SD}}}_{\mathrm{no}\,\mathrm{AA}}^{2}+{{\rm{SD}}}_{{\rm{sample}}}^{2}}$$

### Thiolation assay

Thioester formation of radiolabelled leucine was investigated according to a modified protocol^7^. Enzymes were mixed at 0.5 µM in a total volume of 100 µL TRIS-HCl (pH 7.5) together with 10 mM MgCl_2_, 2 mM TCEP, 2 mM L-Orn, 10 µM L-Leu (980 pmol of non-labelled and 20 pmol of [^3^H]-L-Leu (Hartmann Analytics, Braunschweig, Germany)). The reaction was started by adding 5 mM ATP and quenched after 1 min with ice-cold 800 µL TCA (10% trichloroacetic acid solution). Proteins were coprecipitated with 15 µL ice-cold BSA (25 mg^−1^ml^−1^ solution) and pelleted for 30 min at 13 000 rpm. The pellets were washed twice with 800 µL ice-cold TCA and resuspended in FA (10% formic acid solution). After addition to 3 mL scintillator liquid, the amount of radiolabeled leucine covalently attached to the PPE arm of the respective enzyme was measured in a scintillation counter (Beckman Coulter LS 6500 Liquid Scintillation Counter).

### Product formation followed by mass spectrometry

The formation of the respective peptide products was analyzed by LC/MS. Enzymes (3 µM) were mixed in TRIS-HCl buffer (pH 7.5, final volume of 20 µL) with 10 mM MgCl_2_, 2 mM TCEP, 100 µM L-Val, 100 µM L-Orn and 100 µM L-Leu and incubated at 37 °C. With the addition of 5 mM ATP the reaction was started and after 1 h quenched with 10 µL of formic acid. The samples were pelleted (13 000 rpm, 5 min), the supernatant mixed with 20 µL acetonitrile and 10 µL were subjected to the LC-ESI-MS (Agilent 6130B Single Quadrupole). The MS was used in positive mode together with a NUCLEODUR® HILIC column (27 °C, gradient from 0 to 3 min of 95% B, 3 to 23 min of 95% to 50% B, from 23 to 25 min of 50% B and from 25 to 28 min of 50% to 95% B). Solvent A contained H_2_O, 0.15% FA and 10 mM ammonium formate and B H_2_0, 95% acetonitrile, 0.15% FA and 10 mM ammonium formate. Extracted ion traces of the peptide masses were recorded.

## Supplementary information

Supplementary Information

## Data Availability

Plasmids used and raw data generated in this study are available from the corresponding author upon request.

## References

[1] Stein T (1996). The multiple carrier model of nonribosomal peptide biosynthesis at modular multienzymatic templates. J. Biol. Chem..

[2] Kleinkauf H, Gevers W, Lipmann F (1969). Interrelation Between Activation and Polymerization in Gramicidin S Biosynthesis. Proc. Natl. Acad. Sci..

[3] Quadri LE, Sello J, Keating TA, Weinreb PH, Walsh CT (1998). Identification of a Mycobacterium tuberculosis gene cluster encoding the biosynthetic enzymes for assembly of the virulence-conferring siderophore mycobactin. Chem. Biol..

[4] Felnagle EA (2010). MbtH-Like Proteins as Integral Components of Bacterial Nonribosomal Peptide Synthetases†. Biochemistry.

[5] Lambalot RH (1996). A new enzyme superfamily — the phosphopantetheinyl transferases. Chem. Biol..

[6] De Crécy-Lagard V, Marlière P, Saurin W (1995). Multienzymatic non ribosomal peptide biosynthesis: identification of the functional domains catalysing peptide elongation and epimerisation. Comptes Rendus Académie Sci. Sér. III Sci. Vie.

[7] Stachelhaus T, Mootz HD, Bergendahl V, Marahiel MA (1998). Peptide bond formation in nonribosomal peptide biosynthesis. Catalytic role of the condensation domain. J. Biol. Chem..

[8] Horsman, M. E., Hari, T. P. A. & Boddy, C. N. Polyketide synthase and non-ribosomal peptide synthetase thioesterase selectivity: logic gate or a victim of fate? *Nat*. *Prod*. *Rep*. 10.1039/c4np00148f (2015). 10.1039/c4np00148f 25642666

[9] Awan, A. R., Shaw, W. M. & Ellis, T. Biosynthesis of therapeutic natural products using synthetic biology. *Adv*. *Drug Deliv*. *Rev*, 10.1016/j.addr.2016.04.010 (2016). 10.1016/j.addr.2016.04.010 27094795

[10] Doekel S (2008). Non-ribosomal peptide synthetase module fusions to produce derivatives of daptomycin in Streptomyces roseosporus. Microbiol. Read. Engl..

[11] Bozhüyük KAJ (2018). De novo design and engineering of non-ribosomal peptide synthetases. Nat. Chem..

[12] Bozhüyük, K. A. J. *et al*. Modification and de novo design of non-ribosomal peptide synthetases (NRPS) using specific assembly points within condensation domains. *bioRxiv* 354670, 10.1101/354670 (2018). 10.1038/s41557-019-0276-z 31182822

[13] Reger AS, Carney JM, Gulick AM (2007). Biochemical and crystallographic analysis of substrate binding and conformational changes in acetyl-CoA synthetase. Biochemistry.

[14] Yonus H (2008). Crystal structure of DltA. Implications for the reaction mechanism of non-ribosomal peptide synthetase adenylation domains. J. Biol. Chem..

[15] Du L, He Y, Luo Y (2008). Crystal structure and enantiomer selection by D-alanyl carrier protein ligase DltA from Bacillus cereus. Biochemistry.

[16] Alfermann, J. *et al*. FRET monitoring of a nonribosomal peptide synthetase. *Nat*. *Chem*. *Biol*, 10.1038/nchembio.2435 (2017). 10.1038/nchembio.2435 28759017

[17] Tanovic A, Samel SA, Essen L-O, Marahiel MA (2008). Crystal structure of the termination module of a nonribosomal peptide synthetase. Science.

[18] Bloudoff K, Rodionov D, Schmeing TM (2013). Crystal Structures of the First Condensation Domain of CDA Synthetase Suggest Conformational Changes during the Synthetic Cycle of Nonribosomal Peptide Synthetases. J. Mol. Biol..

[19] Sundlov JA, Shi C, Wilson DJ, Aldrich CC, Gulick AM (2012). Structural and functional investigation of the intermolecular interaction between NRPS adenylation and carrier protein domains. Chem. Biol..

[20] Drake EJ (2016). Structures of two distinct conformations of holo-non-ribosomal peptide synthetases. Nature.

[21] Reimer JM, Aloise MN, Harrison PM, Martin Schmeing T (2016). Synthetic cycle of the initiation module of a formylating nonribosomal peptide synthetase. Nature.

[22] Tarry MJ, Haque AS, Bui KH, Schmeing TM (2017). X-Ray Crystallography and Electron Microscopy of Cross- and Multi-Module Nonribosomal Peptide Synthetase Proteins Reveal a Flexible Architecture. Struct. Lond. Engl. 1993.

[23] Koglin A (2006). Conformational switches modulate protein interactions in peptide antibiotic synthetases. Science.

[24] Frueh DP (2008). Dynamic thiolation–thioesterase structure of a non-ribosomal peptide synthetase. Nature.

[25] Goodrich AC, Harden BJ, Frueh DP (2015). Solution Structure of a Nonribosomal Peptide Synthetase Carrier Protein Loaded with Its Substrate Reveals Transient, Well-Defined Contacts. J. Am. Chem. Soc.

[26] Reimer JM, Haque AS, Tarry MJ, Schmeing TM (2018). Piecing together nonribosomal peptide synthesis. Curr. Opin. Struct. Biol..

[27] Marahiel, M. A. A structural model for multimodular NRPS assembly lines. *Nat*. *Prod*. *Rep*, 10.1039/C5NP00082C (2015). 10.1039/c5np00082c 26429504

[28] Lloyd AJ, Thomann HU, Ibba M, Söll D (1995). A broadly applicable continuous spectrophotometric assay for measuring aminoacyl-tRNA synthetase activity. Nucleic Acids Res..

[29] Wilson DJ, Aldrich CC (2010). A continuous kinetic assay for adenylation enzyme activity and inhibition. Anal. Biochem..

[30] Kittilä T, Schoppet M, Cryle MJ (2016). Online Pyrophosphate Assay for Analyzing Adenylation Domains of Nonribosomal Peptide Synthetases. ChemBioChem..

[31] Hemmerling F, Lebe KE, Wunderlich J, Hahn F (2018). An Unusual Fatty Acyl:Adenylate Ligase (FAAL)–Acyl Carrier Protein (ACP) Didomain in Ambruticin Biosynthesis. ChemBioChem.

[32] Finn RD (2016). The Pfam protein families database: towards a more sustainable future. Nucleic Acids Res..

[33] Mootz HD, Schwarzer D, Marahiel MA (2000). Construction of hybrid peptide synthetases by module and domain fusions. Proc. Natl. Acad. Sci..

[34] Pfeifer BA, Admiraal SJ, Gramajo H, Cane DE, Khosla C (2001). Biosynthesis of complex polyketides in a metabolically engineered strain of E. coli. Science.

[35] Quadri LE (1998). Characterization of Sfp, a Bacillus subtilis phosphopantetheinyl transferase for peptidyl carrier protein domains in peptide synthetases. Biochemistry.

[36] Wilson DJ, Shi C, Teitelbaum AM, Gulick AM, Aldrich CC (2013). Characterization of AusA: A Dimodular Nonribosomal Peptide Synthetase Responsible for the Production of Aureusimine Pyrazinones. Biochemistry.

[37] Bučević‐Popović, V., Šprung, M., Soldo, B. & Pavela‐Vrančič, M. The A9 Core Sequence from NRPS Adenylation Domain Is Relevant for Thioester Formation. *ChemBioChem* **13**, 1913–1920. 10.1002/cbic.201200309 22865785

[38] Marahiel MA, Stachelhaus T, Mootz HD (1997). Modular Peptide Synthetases Involved in Nonribosomal Peptide Synthesis. Chem. Rev..

[39] Gocht M, Marahiel MA (1994). Analysis of core sequences in the D-Phe activating domain of the multifunctional peptide synthetase TycA by site-directed mutagenesis. J. Bacteriol..

[40] Wu R (2008). Mechanism of 4-Chlorobenzoate: Coenzyme A Ligase Catalysis. Biochemistry.

[41] Schlumbohm W (1991). An active serine is involved in covalent substrate amino acid binding at each reaction center of gramicidin S synthetase. J. Biol. Chem..

[42] Gehring AM, Lambalot RH, Vogel KW, Drueckhammer DG, Walsh CT (1997). Ability of Streptomyces spp. aryl carrier proteins and coenzyme A analogs to serve as substrates *in vitro* for E. coli holo-ACP synthase. Chem. Biol..

[43] Mootz HD, Marahiel MA (1997). The tyrocidine biosynthesis operon of Bacillus brevis: complete nucleotide sequence and biochemical characterization of functional internal adenylation domains. J. Bacteriol..

[44] Konz D, Doekel S, Marahiel MA (1999). Molecular and Biochemical Characterization of the Protein Template Controlling Biosynthesis of the Lipopeptide Lichenysin. J. Bacteriol..

[45] Gulick AM (2009). Conformational dynamics in the Acyl-CoA synthetases, adenylation domains of non-ribosomal peptide synthetases, and firefly luciferase. ACS Chem. Biol..

[46] Mitchell CA, Shi C, Aldrich CC, Gulick AM (2012). Structure of PA1221, a Nonribosomal Peptide Synthetase Containing Adenylation and Peptidyl Carrier Protein Domains. Biochemistry.

[47] Meyer S (2016). Biochemical Dissection of the Natural Diversification of Microcystin Provides Lessons for Synthetic Biology of NRPS. Cell Chem. Biol..

[48] Gibson DG (2009). Enzymatic assembly of DNA molecules up to several hundred kilobases. Nat. Methods.

[49] Amann E, Ochs B, Abel KJ (1988). Tightly regulated tac promoter vectors useful for the expression of unfused and fused proteins in Escherichia coli. Gene.

[50] Edelheit O, Hanukoglu A, Hanukoglu I (2009). Simple and efficient site-directed mutagenesis using two single-primer reactions in parallel to generate mutants for protein structure-function studies. BMC Biotechnol..

[51] Hahn M, Stachelhaus T (2004). Selective interaction between nonribosomal peptide synthetases is facilitated by short communication-mediating domains. Proc. Natl. Acad. Sci. USA.

[52] Bergmeyer HU (1975). New values for the molar extinction coefficients of NADH and NADPH for the use in routine laboratories (author’s transl). Z. Klin. Chem. Klin. Biochem..

