# Supplementary material for: Context-dependent activity of A domains in the tyrocidine synthetase
Source: Sci Rep. 2019 Mar 26;9:5119. doi: 10.1038/s41598-019-41492-8 (PMC6435693; doi:10.1038/s41598-019-41492-8)
Supplement: Supplementary file 1 — Supplementary Information [file 41598_2019_41492_MOESM1_ESM.docx]

**Supplementary Information**

**Context-dependent activity of A domains in the tyrocidine synthetase**

Anna Degen^1,4^, Florian Mayerthaler^2^, Henning D. Mootz^2^ and Barbara Di Ventura^3,4*^

1 German Cancer Research Center DKFZ and Faculty of Biosciences, University of Heidelberg, 69120 Heidelberg, Germany

2 Department of Chemistry and Pharmacy, Institute of Biochemistry, University of Münster, 48149 Münster, Germany

3 Institute of Biology II, University of Freiburg, 79104 Freiburg, Germany

4 Signalling Research Centers BIOSS and CIBSS, University of Freiburg, 79104 Freiburg, Germany

[barbara.diventura@biologie.uni-freiburg.de](mailto:barbara.diventura@biologie.uni-freiburg.de)


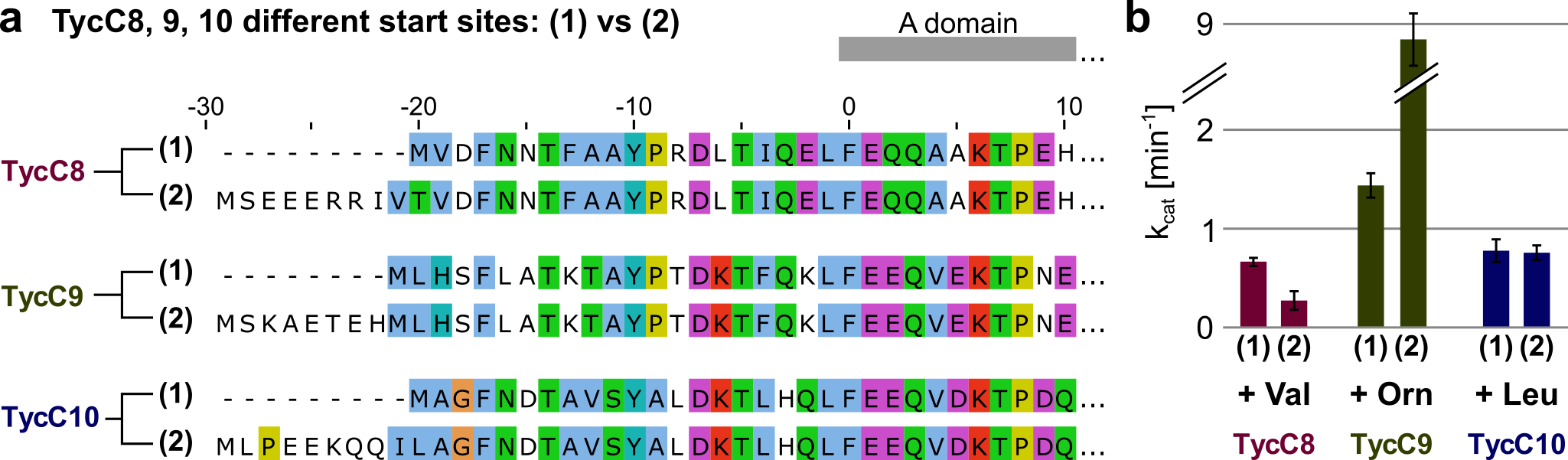


Figure S1| Impact of the start site on activity of A domains within A-PCP di-domain constructs. (a) Sequence alignment of the indicated constructs, where (1) and (2) indicate two different start sites. Amino acids are color-coded according to their category and degree of conservation, following Clustal X coloring scheme. Numbers above the sequence indicate the amino acid position, where the predicted start site of the A domain is set to zero. (b) Bar graph showing k_cat_ values for the indicated constructs in the presence of the indicated amino acids, calculated using the online PP_i_ release assay after subtraction of the background (=no substrate) value. Amino acids were added at 1 mM, enzymes at 0.5 µM. Data represent the mean (± standard deviation, SD) of three independent experiments.


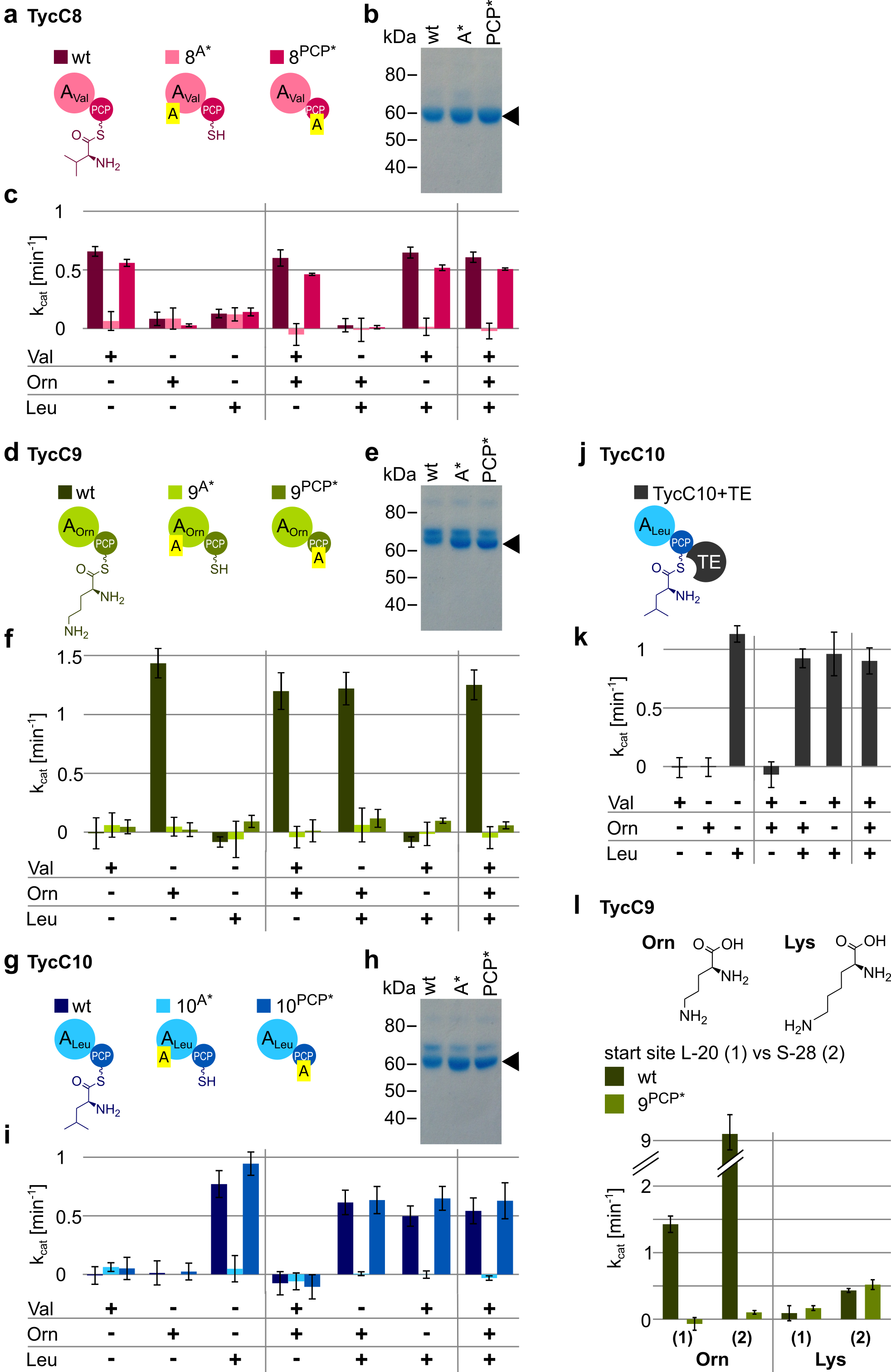


**Figure S2 |** **Activity of selected A domains within “wild type” or mutated A-PCP di-domain constructs of TycC. (a,d,g,k)** Schematic depiction of constructs with amino acids attached to the PPE arm for the wt construct. Colors as in Fig. 1a. **(b,e,h)** Coomassie-stained SDS-gels showing the indicated purified proteins. **(c,f,i,l)** Bar graph showing k_cat_ values for the indicated constructs in the presence of the indicated amino acids, calculated using the online PP_i_ release assay after subtraction of the background (=no substrate) value. Amino acids were added at 1 mM, enzymes at 0.5 µM. Data represent the mean (± standard deviation, SD) of three independent experiments. **(j)** Left: bar graph showing k_cat_ values for the indicated constructs in the presence of the indicated amino acids, calculated using the online PP_i_ release assay after subtraction of the background (=no substrate) value. (1) and (2) indicate the two different start sites shown in Fig. S1a. Amino acids were added at 1 mM, enzymes at 0.5 µM. Data represent the mean (± standard deviation, SD) of three independent experiments. Right: chemical structures of the amino acids used in the assay.


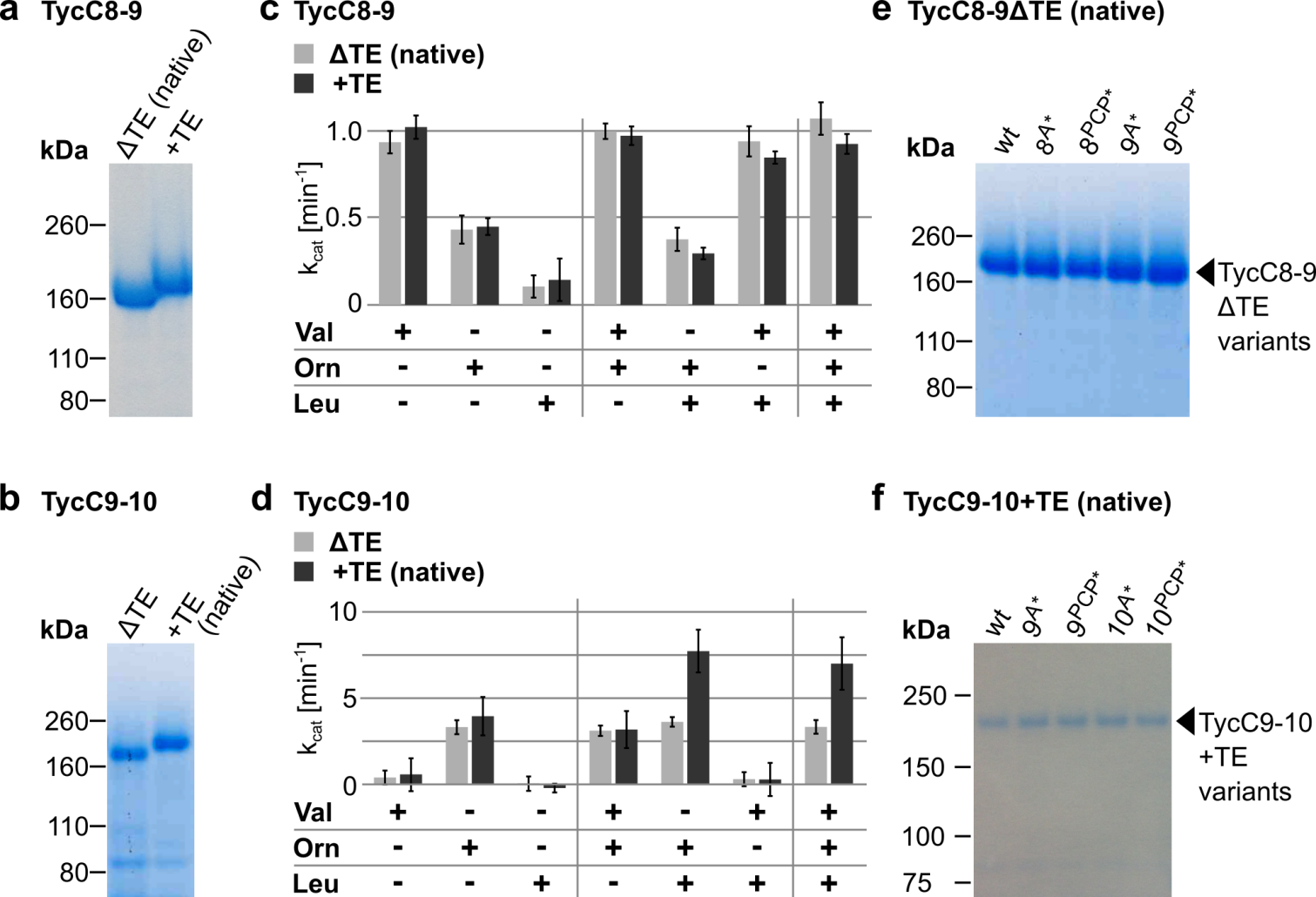


**Figure S3 |** **Activity of selected A domains within “wild type” di-modular constructs of TycC. (a,b and e,f)** Coomassie-stained SDS-gels showing the indicated purified proteins. **(c,d)** Bar graph showing k_cat_ values for the indicated constructs in the presence of the indicated amino acids, calculated using the online PP_i_ release assay after subtraction of the background (=no substrate) value. Amino acids were added at 1 mM, enzymes at 0.5 µM, except TycC9‑10ΔTE, which was added at 0.25 µM. Data represent the mean (± standard deviation, SD) of three independent experiments.

**
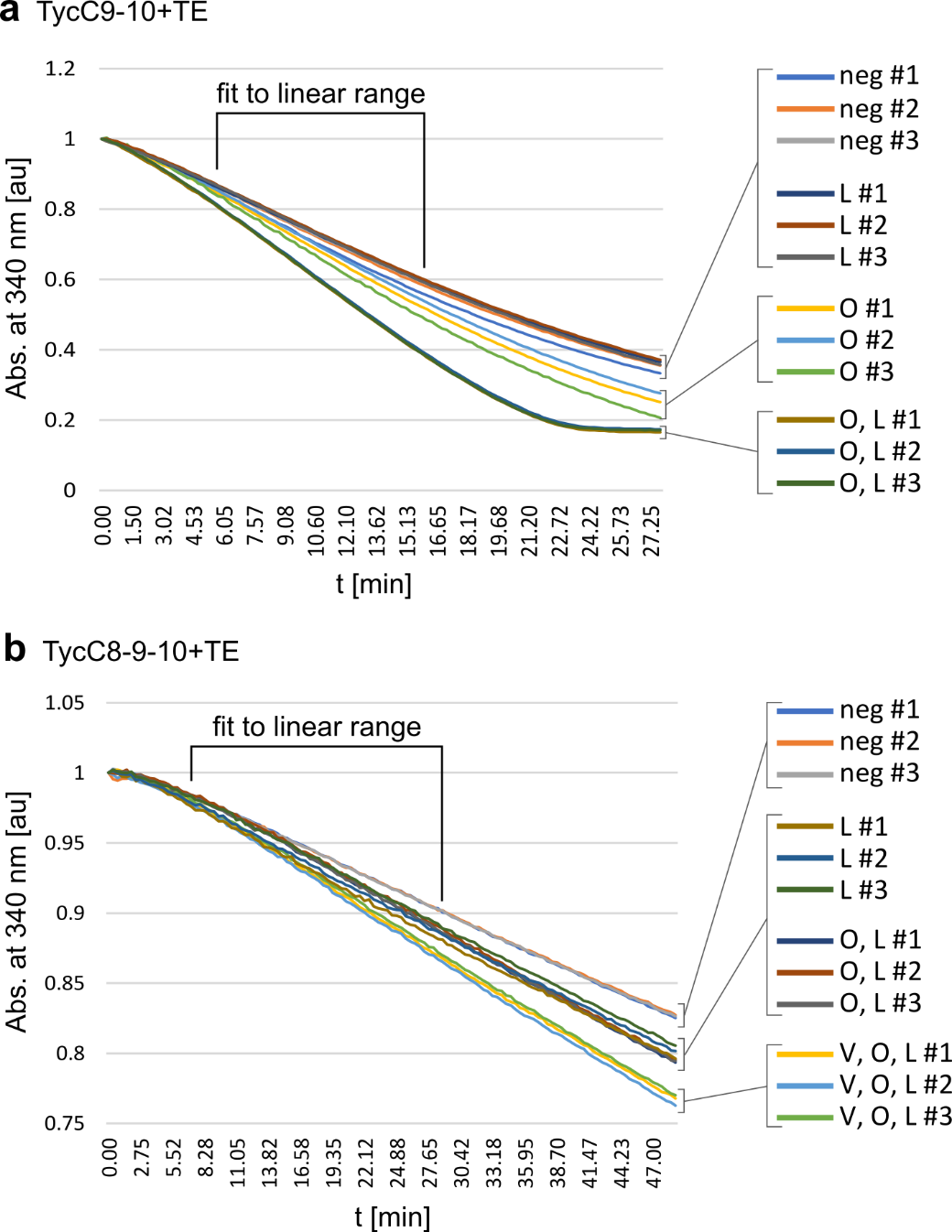
**

**Figure S4 | Representative curves of the PP_i_ release assay. (a,b)** Curves represent the absorption at 340 nm measured over time for the indicated enzymes in different conditions. Neg: negative control (no substrates added to the reaction); L: leucine added to the reaction; O: ornithine added to the reaction; O, L: ornithine and leucine added to the reaction. V, O, L: valine, ornithine and leucine added to the reaction. #: replicate number. A linear fit is used to extrapolate the slope of each curve in the linear range. The slopes are then used to calculate the k_cat_ values, shown in Fig. 4d and Fig. 5c.


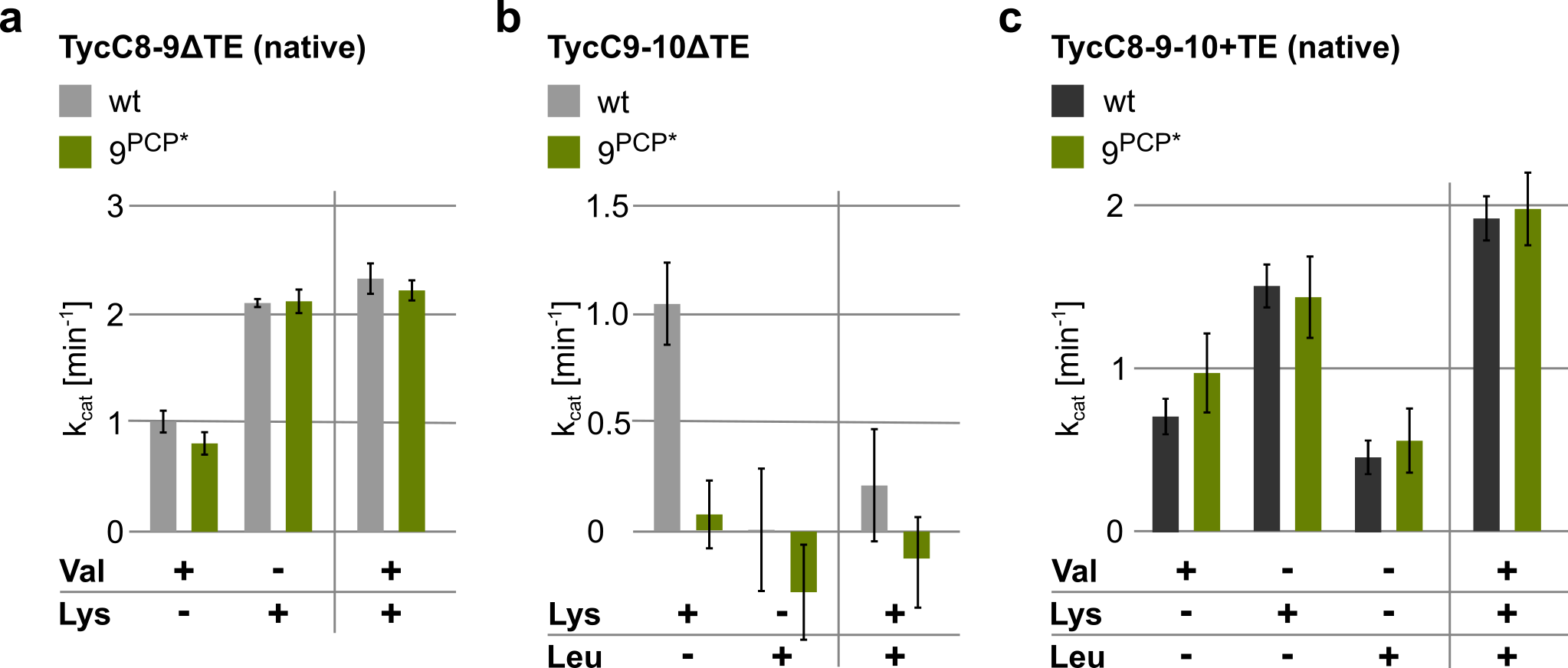


**Figure S5 | Substrate dependent k_cat_ values of different di‑, and tri‑modular TycC constructs when replacing the native substrate Orn by the similar Lys.** **(a-c)** Bar graph showing k_cat_ values for the indicated constructs in the presence of the indicated amino acids, calculated using the online PP_i_ release assay after subtraction of the background (=no substrate) value. Amino acids were added at 1 mM, enzymes at 0.5 µM, except TycC9‑10ΔTE, which was added at 0.25 µM. Data represent the mean (± standard deviation, SD) of three independent experiments.


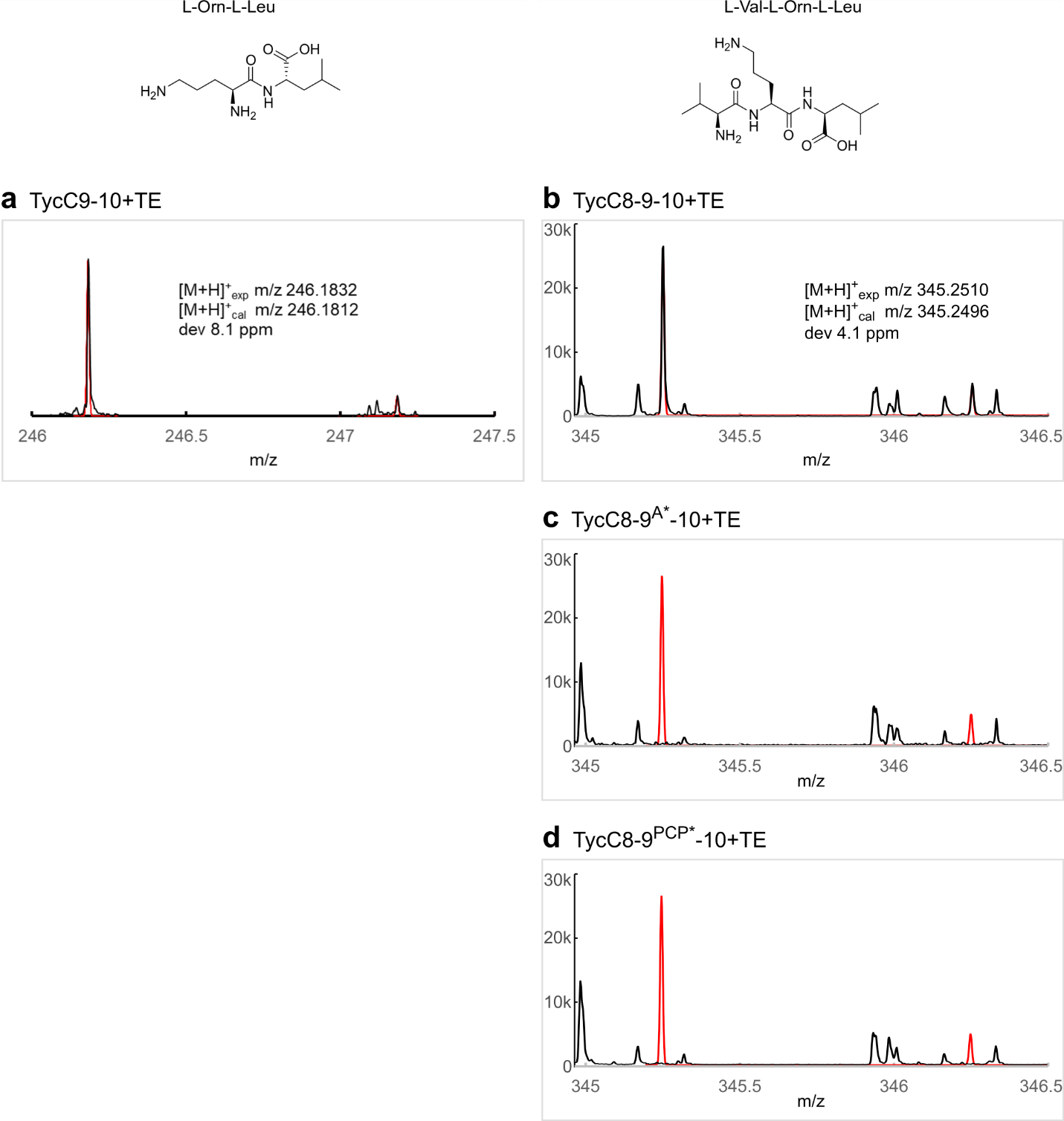


**Figure S6 | Product formation measured by mass spectrometry.**  **(a-d)** MS spectrum of the *in vitro* reaction in which the indicated enzyme was mixed with all substrates to allow peptide production (Orn-Leu (a) and Val-Orn-Leu (b-d)). The black line represents experimental data and the red line a simulation using mMass for the expected signal.

Table S1 | List of primers used in this study

| **TycC insertion into pTrc99a** |  |
| --- | --- |
| 1) TycC(-40V_Ad8)-pTrc_fwd | cacacaggaaacagaccatgGTTGATTTCAACAACACGTTTGCCG |
| 2) TycC(-49S_Ad8)_pTrc_fwd | cacacaggaaacagaccatgAGCGAGGAAGAGCGCCGAATTG |
| TycC(Td8_A4)_rv | gatgatgacctgatcctgcgctctcggcaatatgcg |
| 1) TycC(-41L_Ad9)-pTrc_fwd | cacacaggaaacagaccatgCTGCACAGCTTCCTCGC |
| 2) TycC(-49S_Ad9)_pTrc_fwd | cacacaggaaacagaccatgTCCAAAGCAGAGACGGAGCACATG |
| TycC(Td9_A4)_rv | gatgatgACCTGATCCGGCTGTCTCTTCGATGAACGCC |
| 1) TycC(-40L_Ad10)-pTrc_fwd | cacacaggaaacagaccatgGCCGGGTTCAACGATACGG |
| 2) TycC(-49L_Ad10)_pTrc_fwd | cacacaggaaacagaccatgTTGCCAGAAGAAAAACAGCAGATTTTGG |
| TycC(Td10_R4)_rv | gatgatgACCTGATCCGCGCTTATGCGTGATGAAATCG |
| TycC(Td9_A4)-(-16F_TE)_rv | GCCGTATCTGCTCTCAAAGGCTGTCTCTTCGATGAACGC |
| TycC(-16F_TE)_fwd | TTTGAGAGCAGATACGGCAC |
| TycC(TE)-(GSG-H6)Ptrc_rv | caggctgaaaatcttctctcagtggtgatggtgatgatgACCTGATCCTTTCAGGATGAACAGTTCTTGCAGG |
|  |  |
| TycC(Cd9-10_F278)_fwd | CGTGAACACCTTGGCGATGC |
| TycC(Cd9-10_F278)_rv | GCATCGCCAAGGTGTTCACG |
| TycC(-41L_Ad9)_fwd | CTGCACAGCTTCCTCGCAACC |
| TycC(-41L_Ad9)_rv | GGTTGCGAGGAAGCTGTGCAG |
| **Site-directed mutagenesis** |  |
| TycC-Ad8-D349A_fwd | GATGTATCGCACCGGAGCGTTGGCGAGATGGCTGC |
| TycC-Td8-S28A_fwd | GAAATCGGCGGTCATGCGTTGAAGGCGATGAACG |
| TycC-Ad9-D352A_fwd | GTACCGCACAGGTGCGCTGGCGAAGTGGC |
| TycC-Td9-S28A_fwd | GCTCGGCGGTCACGCTTTGCGTGCCATGC |
| TycC-Ad10-D354A_fwd | CATGTACAAAACAGGCGCGTTGGTAAAATGGCGGAC |
| TycC-Td10-S29A_fwd | GAACTCGGAGGACATGCCTTAAAAGCTACGC |
| **Sequencing** |  |
| ptrc99a seq_fwd | GCGCCGACATCATAACGGTTC |
| ptrc99a seq_rv | CGCTACTGCCGCCAGGC |
| TycC-Ad8(L239)_fwd | GTCGCCGAAGCACATCAACC |
| TycC-Ad9(E135)_fwd | GAGCACCGCAGCTATGCC |
| TycC-Ad9(P296)_fwd | CGCTTCCGAACATGACGATG |
| TycC-Ad10(E61)_fwd | GAGCGCATTCAGTACCTGCTC |
| TycC-Ad10(Q234)_fwd | GTTGCGTTGCGTCACTTTGG |
| **Backbone amplification** |  |
| pTrc99_Orf_fwd | tgagagaagattttcagcctg |
| pTrc99_Orf_rv | catggtctgtttcctgtgtg |
| pTrc99-GSG-H6_fwd | GGATCAGGTcatcatcacc |
